# Gianotti-Crosti Syndrome: A Benign Dermatosis

**DOI:** 10.7759/cureus.40328

**Published:** 2023-06-12

**Authors:** Victor N Oboli, Imoh L Ebong, Omaira Tejada Amaro, Jefferson A Regis, Muhammad Waseem

**Affiliations:** 1 Pediatrics, NYC (New York City) Health + Hospitals/Lincoln, New York, USA; 2 Pediatrics, NYC (New York City) Health + Hospitals/Woodhull, New York, USA; 3 School of Medicine, St. George's University School of Medicine, St. George's, GRD; 4 Pediatrics and Emergency Medicine, NYC (New York City) Health + Hospitals/Lincoln, New York, USA

**Keywords:** generalised skin rash, mmr vaccine, type iv hypersensitivity reaction, maculo-papular rash, gianotti-crosti syndrome, immunization, acral dermatitis

## Abstract

Gianotti-Crosti syndrome (GCS) is a benign acral dermatitis commonly seen in children younger than five years of age with no gender predilection. Clinical features are often vague, including but not limited to fever, lymphadenopathy, and erythematous papular rash that commonly spares the trunk, palms, and soles of the feet. It is presumably underdiagnosed as most children presenting with a widespread papular rash are diagnosed with non-specific viral exanthem. This benign condition has been linked to multiple viruses, and treatment is mainly supportive. We present a previously healthy 18-month-old girl who presented to the emergency room with a progressive skin rash and low-grade fever 10 days after receiving routine immunizations. GCS was diagnosed, and she received supportive care with spontaneous resolution of symptoms in four weeks.

## Introduction

Gianotti-Crosti syndrome (GCS), also called acral dermatitis of childhood, is a benign and self-limiting exanthem manifesting as papular or papulovesicular lesions [1-3]. The rash has a characteristic symmetric, monomorphic, and erythematous appearance measuring about 1 to 10 mm in diameter with a tendency to coalesce into plaques. It is non-relapsing, with paracortical lymph node hyperplasia and anicteric acute hepatitis. However, it is believed that neither lymphadenopathy nor hepatitis is required for this diagnosis [4,5].

## Case presentation

**Figure 1:**
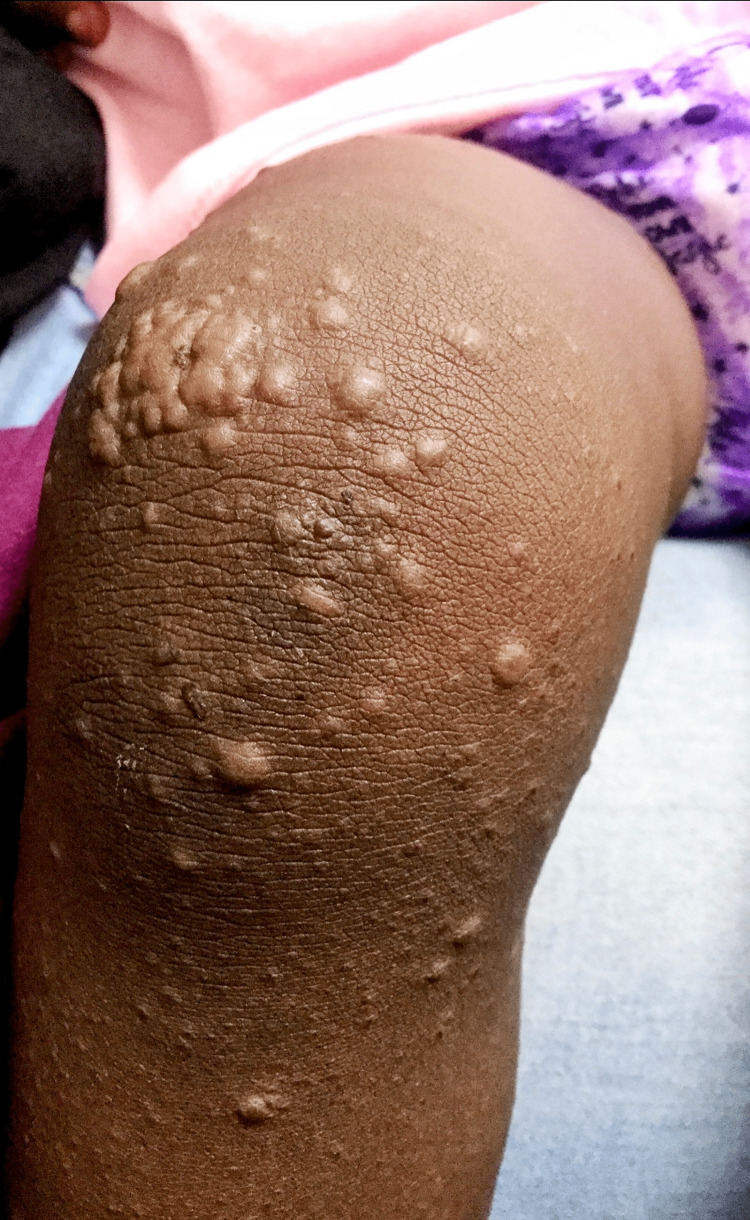
Anterior knee showing multiple maculopapular lesions measuring about 5-10 mm in diameter and with few scarifications marks.

**Figure 2:**
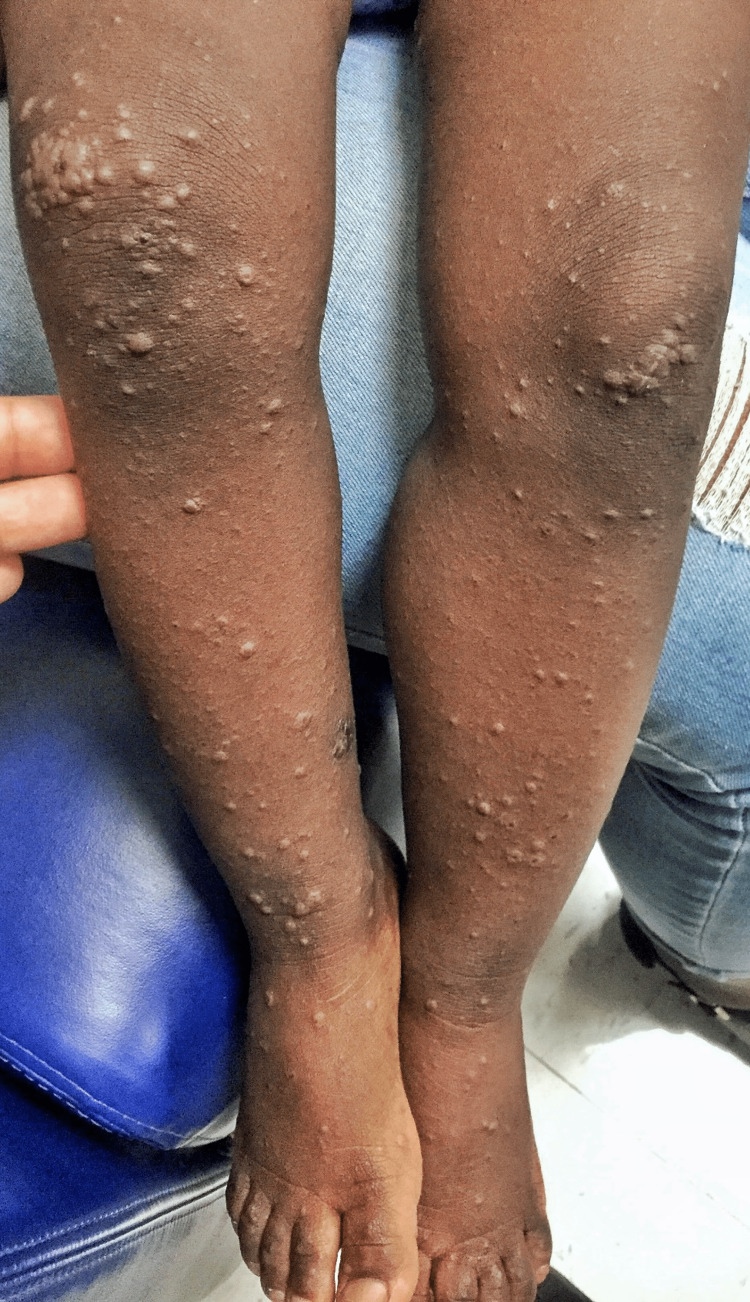
Bilateral anterior legs showing multiple isolated and coalesced maculopapular lesions measuring about 5-10 mm in diameter.

**Figure 3:**
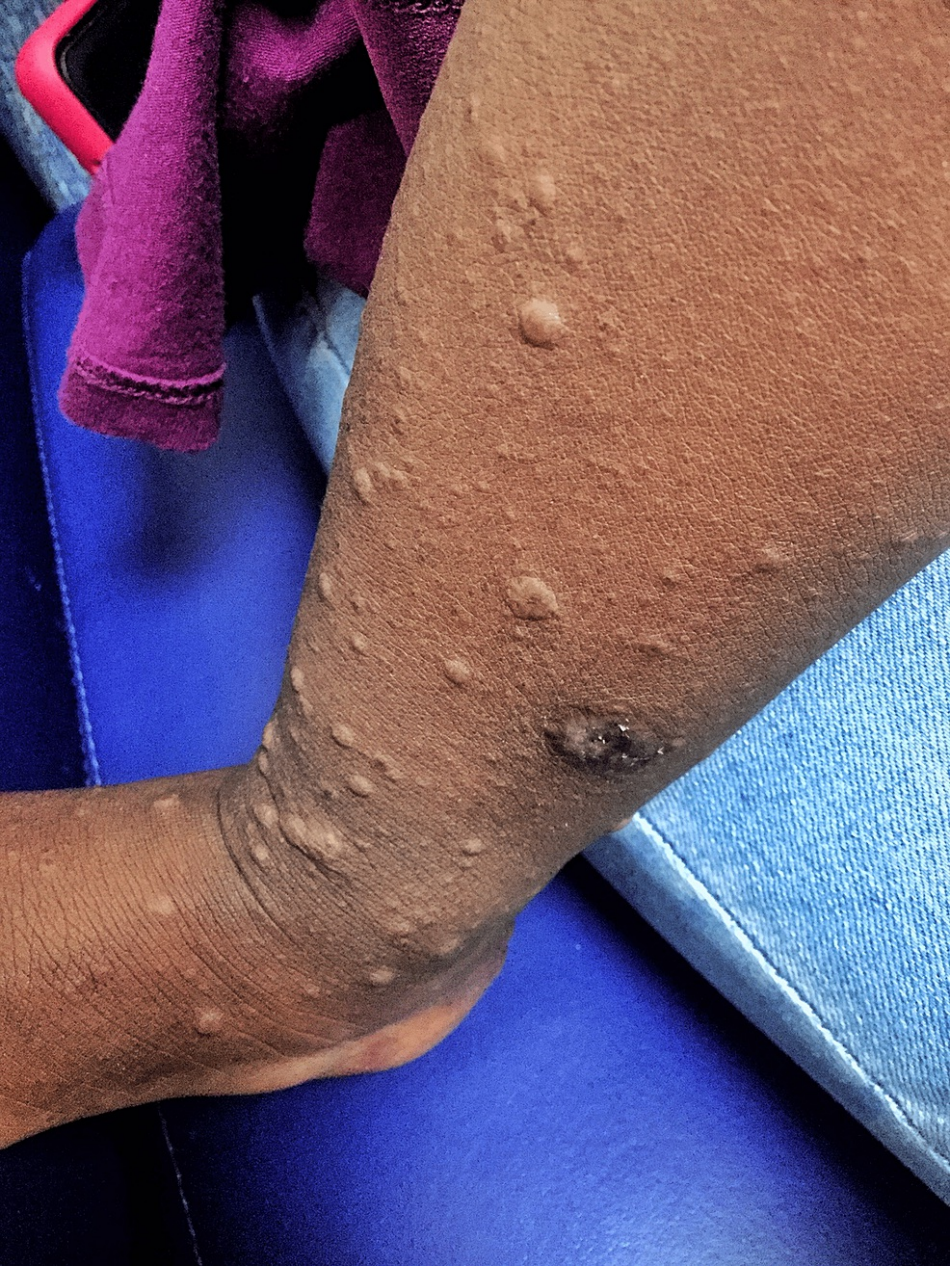
Right anterior leg showing multiple isolated papular lesions measuring about 5-7 mm in diameter with sparse areas of hyperpigmentation.

A previously healthy 18-month-old girl was brought to the emergency department (ED) with a two-day history of low-grade fever and skin rash involving all the limbs and face. The rash began on the lower extremities and progressed to involve the upper limbs and face, sparing the palms of the hand and soles of the feet. The mother denied itchiness, pain, cough, rhinorrhea, malaise, sick contacts, recent travel, or medication use. The child received immunizations against hepatitis A, inactivated poliovirus, measles, mumps, rubella, influenza, and hepatitis B eight days before the onset of the rash. On arrival, vital signs were temperature of 100.1 F, heart rate - 104/minute, respiratory rate - 26/minute, blood pressure - 79/50 mm Hg, and oxygen saturation of 99% in ambient room air. Physical examination was notable for several widespread maculopapular lesions on the anterior knees and legs (Figures 1, 2, 3), face, and upper extremities measuring about 5-10 mm in diameter. There were no palpable regional lymph nodes. The remainder of her examination was non-contributory. Viral panel testing was negative. The child improved with supportive care, and the rash resolved.

## Discussion

The clinical features of GCS are non-specific and often mimic viral exanthems. GCS affects males and females equally, with a 1:1 ratio [1]. The symptoms associated with GCS may include low-grade fever, lymphadenopathy, and an erythematous papular confluent pruritic rash. The rash primarily involves the cheeks of the face, extensor surfaces of the limbs, and the buttocks. In most cases, the rash spares the trunk, palms, and soles of the feet [6]. Several studies have demonstrated associations between GCS and viral infections, with Epstein-Barr virus (EBV) and Hepatitis B virus (HBV) being most commonly implicated [2,3,7]. These viruses are potentially dangerous and should be considered when evaluating children with GCS. Other associations include the onset of GCS after vaccinations against influenza and measles-mumps-rubella (MMR) viruses [8,9]. A case of GCS following vaccination against Japanese B encephalitis was reported by Kang et al. [10]. Some bacterial infections, such as *Mycoplasma pneumoniae*, beta-hemolytic Streptococci species, and *Borrelia burgdorferi*, have been linked to GCS [11,12]. Perhaps, vaccination was the most likely trigger in this case. Magyarlaki et al. proposed that the rash erupts from a pathogen-induced type IV cutaneous hypersensitivity reaction secondary to inflammatory infiltrate activity [13]. GCS can also present without an apparent prodrome [3]. Diagnosis of GCS is mainly clinical. A detailed history and physical examination are mandatory for diagnosing GCS. Routine investigations are not required, especially if the patient is clinically stable without abnormal systemic findings [14]. The rash of GCS can be discomforting and itchy, and treatment is primarily supportive. Anti-histamines can be administered to alleviate itching. Oral antipyretics can be given for fever control. Skin lesions tend to resolve spontaneously in 2-8 weeks. In addition, it is essential to provide patient education and reassurance to the family, highlighting the self-limiting nature of the condition without a contraindication to childhood immunization. Finally, close follow-up appointments are required to monitor the lesions and address parental concerns.

## Conclusions

This case highlights the importance of considering GCS as a differential diagnosis when evaluating children presenting with a papular rash. In addition, the case underscores the need for awareness and knowledge of this condition to ensure accurate diagnosis and appropriate management strategies. By gaining a deeper understanding of GCS, pediatricians can effectively identify this rare skin condition, provide the necessary care to affected children, avoid unnecessary investigations in subsequent cases, and prevent vaccine restrictions.
